# Invasive Lobular Carcinoma Arising in Ectopic Breast Tissue: A Case Report

**DOI:** 10.7759/cureus.24055

**Published:** 2022-04-12

**Authors:** Joana Marques-Antunes, Florinda Cardoso, Teresa Santos, Mário Nora, Horácio Scigliano

**Affiliations:** 1 General Surgery, Centro Hospitalar de Entre Douro e Vouga, Santa Maria da Feira, PRT; 2 Pathology, Centro Hospitalar de Entre Douro e Vouga, Santa Maria da Feira, PRT

**Keywords:** ectopic breast tissue, polythelia, axilla, lobular carcinoma, breast cancer

## Abstract

Incomplete regression of the embryonic mammary line occurs in 0.3-6% of the population. Ectopic breast tissue is mostly asymptomatic and can undergo malignant transformation. Ectopic breast cancer accounts for 0.2-0.6% of all breast cancers. Screening breast examinations can miss these lesions due to their location making the diagnosis more challenging. We describe a case of a primary invasive lobular carcinoma in an ectopic breast on the left axilla detected in a 49-year-old woman. Firstly diagnosed as a sebaceous cyst, the lesion was excised under local anesthesia. Histopathology showed breast tissue widely infiltrated by an invasive carcinoma. Excision of the remnant tissue with axillary lymph node dissection was performed. Ectopic breast carcinoma is a rare diagnosis and there is a general lack of awareness. The presence of an abnormal mass along the mammary ridge should raise clinicians’ attention. Management of primary ectopic breast carcinoma should be based on a multidisciplinary approach under the same principles as breast cancer. Furthermore, it does not appear to bring a worse prognosis when diagnosed at similar disease stages.

## Introduction

The presence of ectopic or supernumerary breast tissue is rare, with a reported incidence of 0.3-6% [1]. During the 4th week of pregnancy, embryonic breast development starts. On the ventral surface of the body, bilateral mammary ectodermal tissue forms a ridge, called milk line, extending from the base of the future axilla to the inguinal region. In the 5th week, the mammary ridge disappears gradually, except at the 4th intercostal space, where cells continue to proliferate and differentiate into mammary lobules [2]. Failure of this involution can lead to ectopic breast tissue presenting as polymastia (accessory mammary glands) or polythelia (accessory nipples) [3]. Ectopic breast tissue is mostly asymptomatic. It is identical to normal anatomic breast tissue and vulnerable to the same disorders including malignant transformation [4].

Ectopic breast cancer accounts for 0.2-0.6% of all breast cancers [5]. The axilla is most frequently involved (70-90%) and the glandular tissue is located in the subcutaneous tissue and deep dermis [6-7]. Differential diagnoses from other subcutaneous masses arising in this location include lymphadenopathy, sebaceous cysts, nevus, lipoma, or hidradenitis [4]. Due to its rarity, the diagnosis is commonly delayed, with a calculated delay of 40.5 months on average [8]. The development of breast cancer in atypical locations produces both diagnostic and surgical challenges. The outcome of accessory breast cancer is thought to be poor due to its rarity, early lymph nodes involvement, and late diagnosis [5].

## Case presentation

Medical history

A 49-year-old premenopausal nulliparous woman presented with a palpable lump in her left axilla. She had a congenital single kidney and no history of breast cancer in the family. It was a slow-growing palpable mass causing occasional discomfort with no other associated symptoms. On clinical examination, she had a 1 cm regular mass in the right axilla and no palpable breast lumps. There was no past estrogen or oral contraceptive intake.

Investigation

Ultrasound reported a thickness of subcutaneous cellular tissue, suggestive of a sebaceous cyst, with 13 mm, without suspicious findings or axillary adenopathy. The last bilateral mammogram was performed a year before and was normal. Suspected ectopic breast tissue has never been described previously, nor the patient has ever perceived any axillary lump before. Excision of the cyst was performed under local anesthesia. Histopathology showed ectopic breast tissue infiltrated by invasive lobular carcinoma with 13 mm in size and intersected by margins. The immunohistochemical study had positive estrogen and progesterone receptors, 100% and 95%, respectively, negative human epidermal growth factor receptor 2 (HER2) expression and the Ki67 proliferation index was 3%. No lymph node structures were identified. Given the unexpected results, MRI was performed. No lumps or suspicious areas were seen in any of the breasts. There was no axillary or internal mammary adenopathy (breast imaging reporting and data system (BIRADS 2)).

Treatment

**Figure 1:**
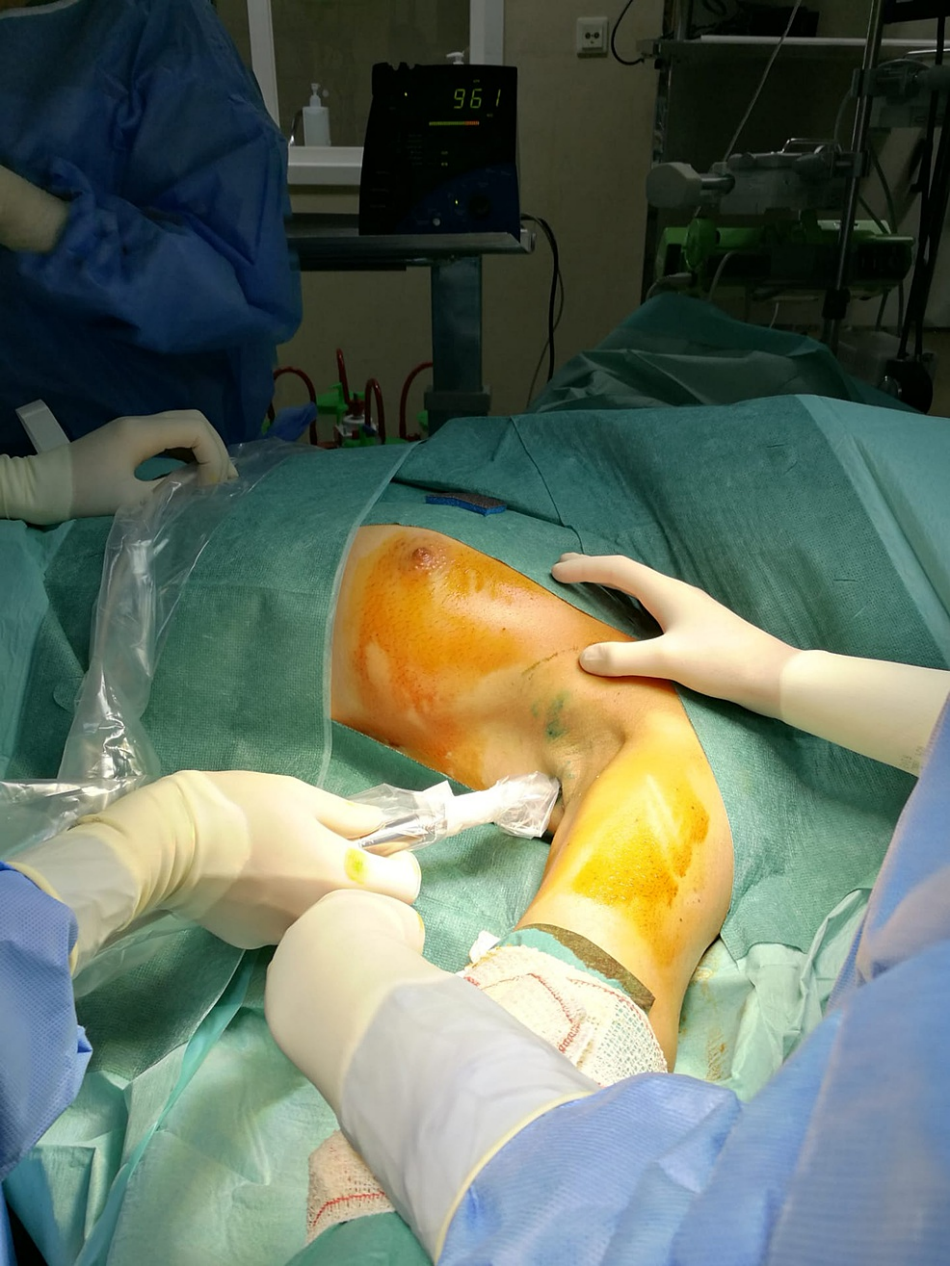
Sentinel lymph node identification protocol with gamma probe and blue dye (Dual technique).

After evaluation by a multidisciplinary board, the patient was proposed for an extended excision of the surgical scar and sentinel lymph node biopsy. At our institution, in eutopic breast cancer, we address axilla in conformity to ACOSG Z0011 (Alliance) protocol [9]. Axillary lymph node dissection is laid aside when only one or two positive lymph nodes are identified. The patient was submitted to excision of the remnant ectopic breast tissue with sentinel lymph node biopsy using a dual technique protocol with gamma probe and blue dye injection (Figure 1).

**Figure 2:**
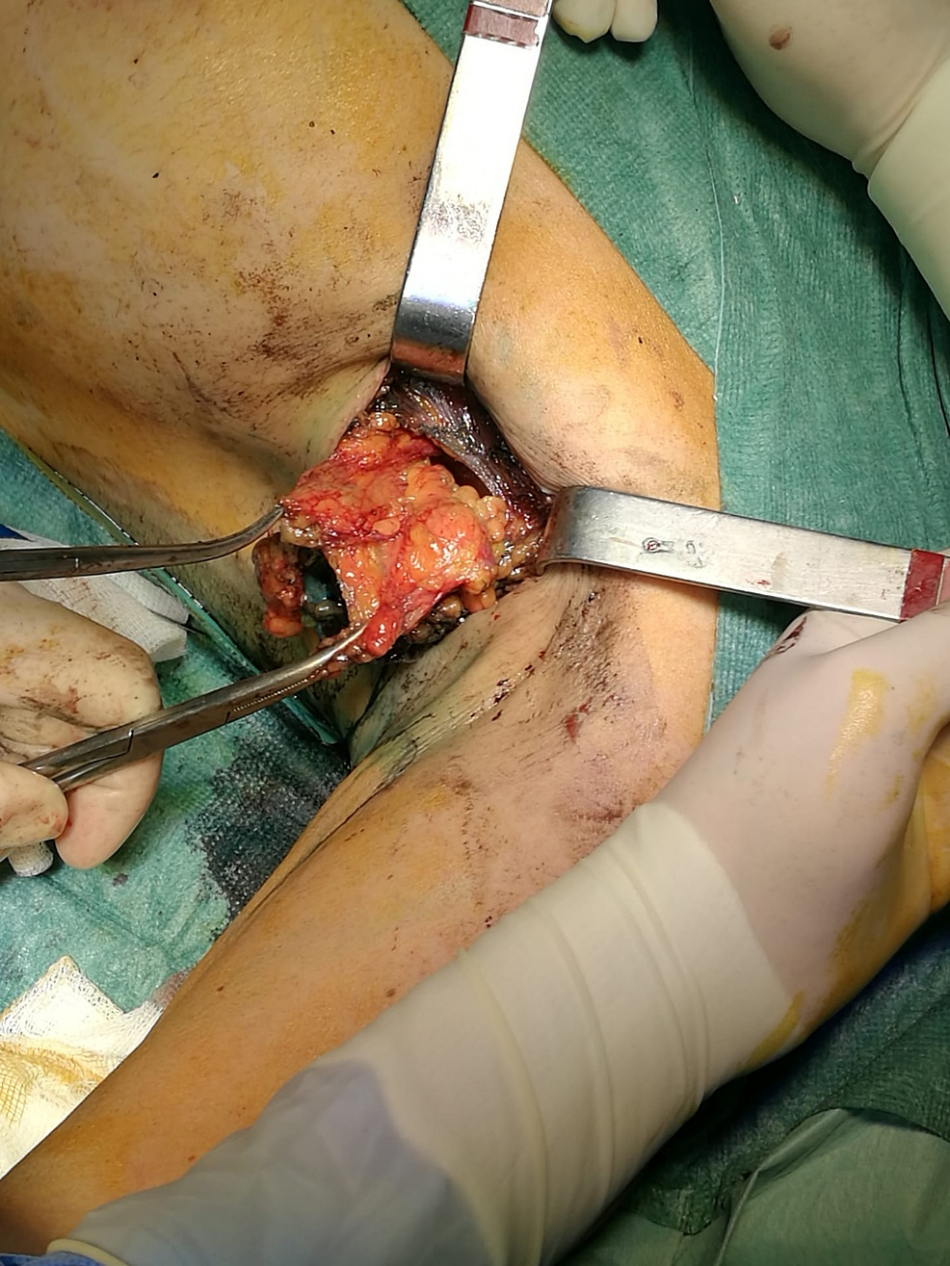
Extended excision with axillary lymph node dissection.

Blue dye injection was performed around the tumor. Probably due to the inflammatory process of the recent manipulation, the tissue had multiple lymph nodes and was too much stained. Sentinel node identification was impossible and axillary lymph node dissection was performed (Figure 2).

**Figure 3:**
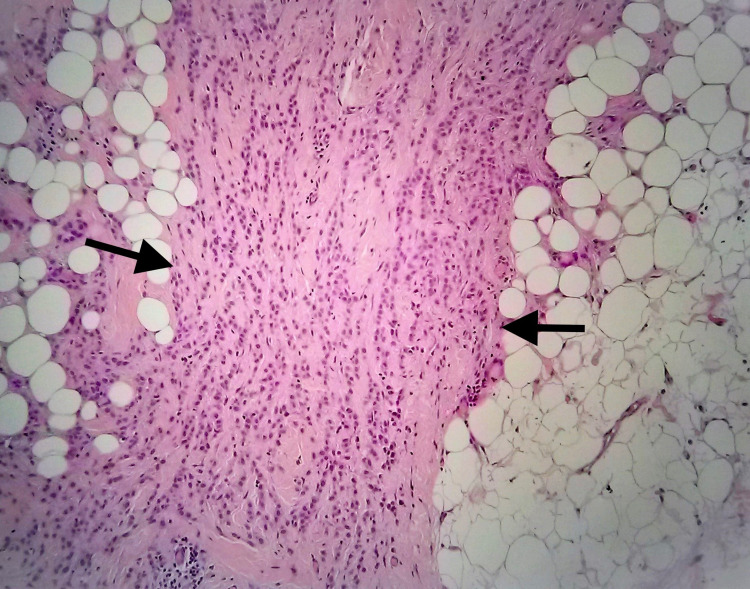
Axillary region. Typical single files of invasive lobular carcinoma (black arrows; 4×; hematoxylin and eosin (H&E))

**Figure 4:**
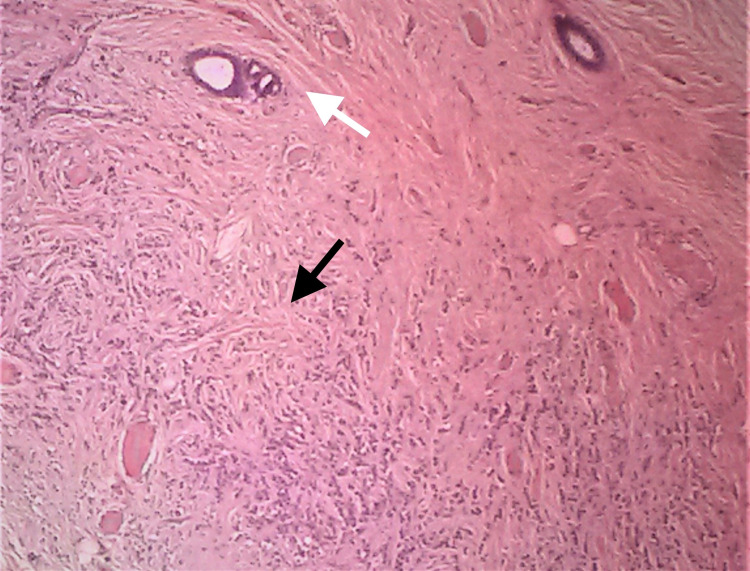
Closeup view of classic invasive lobular carcinoma in the ectopic axillary breast. Descohesive tumor cells arranged in single files or cords. (white arrow normal; black arrow abnormal; 400×; hematoxylin and eosin (H&E))

Final histopathology revealed 6 mm foci of invasive lobular carcinoma and seven nonmetastatic lymph nodes (Figures 3-4).

The tumor was 26 mm away from the nearest margin and the skin was free of tumor. Pathologically it was staged as pT1N0M0 according to Pathological Tumor-Node-Metastasis (pTNM), the American Joint Committee on Cancer (AJCC) 8th edition. The patient underwent radiotherapy to the right breast and axilla and hormonal treatment. Through a 6 monthly follow-up, in the last 2 years, she had no evidence of recurrence or postoperative complications.

## Discussion

Ectopic breast cancer diagnosis and management follow the same assumptions as breast cancer. Due to its rarity, there is no available evidence to sustain a different approach. It is required a high suspicion index to diagnose a carcinoma arising from an ectopic breast. Given its location and the absence of a nipple-areola complex, these lesions are frequently first diagnosed as lipomas or adenopathy [10]. The approach to suspected abnormal ectopic breast tissue follows the same principles as eutopic tissue. Anamnesis, physical examination, and complementary diagnostic tests are indicated whenever there is a suspicious mass in the axilla or along the milk line [11]. Routine mammograms can miss accessory breast tissue due to its location. Ultrasound should complement mammography. MRI could be performed to exclude ipsilateral or contralateral breast carcinoma. Since ectopic breast tissue can be bilateral, contralateral evaluation should be strongly considered [7]. A biopsy is indicated whenever there is a suspicious mass with any worrisome signs in the imaging study. In this case, because it’s lobular carcinoma, MRI was recommended [12]. The incidence of lobular histologic subtype among ectopic breast carcinoma is reported from 3 to 9.5% [6] [13]. Histologically, the lack of lymph node tissue and the presence of adjacent normal breast ducts and lobules are required to confirm the diagnosis of ectopic breast carcinoma and to exclude the possibility of a metastatic tumor arising from an occult primary lesion [14]. Ectopic breast cancer is classified according to the TNM staging system for primary breast carcinoma. As a consequence of its subcutaneous localization, the skin involvement could be premature, staging cancer as T4 [6]. Like other axillary lesions, lymph drains into axillary nodes.

Due to its rarity and paucity of data, surgical management has been a little controversial but has been evolving through the last few years. Historically, ectopic breast cancer was treated with excision of the ectopic breast tissue, radical mastectomy, and chest irradiation [15]. Later, Cogswell and Czerny documented that ipsilateral mastectomy in these patients does not result in a more suitable prognosis. In a series of autopsied patients with ectopic breast cancer that died of metastatic lesions in the skeletal system, pleura, and suprarenal bodies, there was no evidence of infiltration to the ipsilateral breast of the primary tumor. No breast involvement had been seen on surgical specimens of radical mastectomy in patients with distant metastases. Moreover, early recurrence was described after radical mastectomy, as well as, after wide local excision [16]. Evans and Guyton concluded that there was no additional advantage of mastectomy over local excision and axillary lymph node dissection [7]. These aggressive technics are no longer an option if there is not a malignant lesion at the breast. More recently, local excision is the procedure recommended and mastectomy can be considered only in those patients who have an additional lesion in the breast. We believe that early stage cancer can be treated with wide local excision, sentinel lymph node biopsy, and axillary dissection if required. Axillary lymph node dissection is done only if lymph nodes are involved. When the blue dye is used, an intradermal injection may be required because of the subdermal nature of the lesion in the axilla [17]. In our case, the injection into the axilla stained much of the tissue and made sentinel node identification difficult. The principles of adjuvant treatment are the same as for anatomic breast cancer.

Regarding adjuvant treatments, patients with hormone-positive receptors should be offered adjuvant hormone therapy because it reduces contralateral breast cancer and recurrence rate [18]. Postoperative radiotherapy is also indicated to minimize the risk of locoregional recurrence [19]. Patients should be prescribed chemotherapy depending on the tumor characteristics according to standard breast cancer guidelines.

The prognosis of patients with ectopic breast carcinoma is difficult to establish because of the limited follow-up data and small sample size reports. In a review article by Marshall et al., poorer outcomes in ectopic breast cancer were imputed to clinical management instead of the disease itself [13]. Nihon-Yanagi et al. showed that the correlation between T stage and lymph node metastasis suggested that not all ectopic breast cancers arising in the axilla have a higher risk of nodal metastases than usual breast cancer. Ectopic breast cancer, therefore, does not appear to carry a worse prognosis than usual breast cancer, when diagnosed at similar disease stages [6]. The poorer prognosis than cancer developed in normal breast parenchyma can be due to diagnosis delay. There are no specific guidelines for surveillance and regular follow-up is recommended [8].

## Conclusions

Ectopic breast carcinoma is a rare diagnosis and there is a general lack of awareness among clinicians. Furthermore, accessory axillary breast tissue is left apart in the majority of screening breast examinations. Management of primary ectopic breast carcinoma should be based on a multidisciplinary approach under the same principles as breast cancer. Ectopic breast cancer does not appear to bring a worse prognosis when diagnosed at similar disease stages.
